# Introduction to the supplement

**DOI:** 10.1007/s00787-012-0353-y

**Published:** 2012-12-04

**Authors:** Hans-Christoph Steinhausen

**Affiliations:** 1 Research Unit for Child and Adolescent Psychiatry, Aalborg Psychiatric Hospital, Aarhus University Hospital, Mølleparkvej 10, 9000 Aalborg, Denmark; 2 Clinical Psychology and Epidemiology, Institute of Psychology, University of Basel, Missionsstrasse 60/62, 4055 Basel, Switzerland; 3 Department of Child and Adolescent Psychiatry, University of Zurich, Neptunstrasse 60, 8032 Zürich, Switzerland

The concepts of child and adolescent psychiatric disorders show both continuity and change. Whereas the main broad psychopathological phenomena do not change fundamentally, there is an ongoing process of refining diagnostic criteria of various disorders, delineating new subtypes, and, from time to time, also the definition of a new diagnostic entity. These processes are reflected in the major classification systems forming an important framework of clinical work and service delivery which are existing now already for several decades. Another important cornerstone structuring clinical practice is the increasing number of disorder-specific guidelines for both assessment and treatment of these disorders.

Currently, the revision of the two major international classification systems, the US-American Diagnostic and Statistical Manual of Mental Disorders, fifth revision (DSM-5) and the International Classification of Diseases, 11th revision are in a relatively advanced stage of development with the DSM-5 expected to be published in May 2013 and the ICD-11 2 years later in 2015. Irrespective of the evaluation whether the upcoming changes in these classification of child and adolescent disorders are mainly driven by research findings or by political or even financial interests, it is clear that these revisions will have a strong impact on the understanding of these disorders both in clinical care and research.

Thus, it is time to make clinicians and service providers acquainted with the current status of the upcoming changes in the classification of the major mental disorders in children and adolescents. Currently, there is much more information available on the upcoming changes in the DSM than in the ICD. However, the cross-talk between the two systems, certainly, will have a strong impact for the 11th revision of the ICD. In the first part, the chapters on the various disorders concentrate on the question what kind of changes we will have to expect in the DSM-5 and what kind of impact these changes will have for clinical practice.

The present collection of papers is restricted to the major mental disorders in childhood and adolescence. Many of them continue into adulthood and could be regarded as a specific manifestation at a young age so that one may indeed argue that a separate grouping as disorders with an onset specific to childhood could be deleted from the upcoming revised classifications. This pertains, e.g., for the schizophrenic psychoses, the affective disorders, the anxiety disorders, and the obsessive–compulsive disorders.

However, other disorders have a rather characteristic onset early in life. They include the infant psychiatric disorders, the autism spectrum disorders, and the elimination disorders which are discussed in the present supplement. All of these disorders will either show full remission in childhood or only minor or moderate transformations of symptoms but lasting effects on psychosocial functioning for the entire life like the autism spectrum disorders. In the DSM-5, some of these disorders will be reclassified as neurodevelopmental disorders including also the very common attention-deficit–hyperactivity disorders, or as a separate category like the elimination disorders. Similarly, another very common disorder in childhood and adolescence, namely, conduct disorder will also be reclassified in a new category of disruptive, impulse control, and conduct disorders rather than keeping it within a group of disorders with onset in infancy, childhood, and adolescence. Furthermore, revised classifications may include candidates of new disorders with a known clinical manifestation but perhaps still lacking sufficient proof of validity. Currently, the non-suicidal self-injury is such a new disorder in DSM-5 which, however, is only listed in a final class with the unspecific name of “Other disorders”.

In addition to introducing the upcoming changes in DSM-5, each chapter of the present supplement includes a brief review of the impact of clinical guidelines for the various disorders. The main aim of clinical guidelines is to assure high-quality assessment and to make the most advanced treatment options available to the patient. According to the British National Institute of Health and Clinical Excellence (NICE) in the National Health System, recommendations are based on the best available empirical evidence, include the views of experts, patients and carers, are developed by independent and unbiased advisory committees, are staying up-to-date by regular reviews, are based on an open and transparent consultation process, and value the input of patients, carers and the general public.

In the recent past, there has been an increasing trend in international child and adolescent psychiatry to design and publish guidelines for a large variety of clinical disorders and problems. Again, this process is most advanced in the USA with a substantial number of practice parameters authored by expert committees and edited by the American Academy of Child and Adolescent Psychiatry (AACAP). The parameters are either patient oriented or clinician oriented. The former are aiming at providing clinicians with assessment and treatment recommendations for child and adolescent disorders whereas the latter inform clinicians about principles of the assessment of children, adolescents, and their families, and the management of children and adolescents with special mental health needs. All these parameters have been published in the journal of the AACAP and are available via the internet of this association.

So far, there has been undertaken no comparable effort to design a similar number of child and adolescent psychiatric guidelines in any other part of the world. European efforts have pertained to the design of a few guidelines addressing hyperkinetic disorders/ADHD, anorexia nervosa, depression, and tic disorders. All these guidelines have been published in this journal, but, due to the lack of initiatives by a central coordinating professional association, are not available on the internet. In addition, national child and adolescent psychiatric guidelines have been produced in various countries.

Clinical guidelines should unfold a strong impact on clinical practice if they are reflected and implemented in practice. Currently, it is not well studied to which extent clinicians actually adhere to guidelines. A first step for their implementation is sufficient information and advice. Accordingly, each chapter of the present supplement introduces the most relevant guidelines for assessment and treatment of the various child and adolescent psychiatric disorders and provides some suggestions how the guideline recommendations may be successfully implemented into clinical care. However, it should also be clear that guidelines need to be adjusted to the needs of the individual patient. Whereas on the one hand there should be no room for ignorance and uncritical beliefs in clinical practice, there should also be a place left for clinical expertise and wisdom that may not be adequately covered by any guideline.

